# A Rare Case of Pulmonary Emboli Presenting With ST Elevation on ECG

**DOI:** 10.7759/cureus.29249

**Published:** 2022-09-16

**Authors:** Zaid Gheith, Bilal Alqam, Rajani Jagana

**Affiliations:** 1 Internal Medicine, University of Kansas Medical Center, Kansas City, USA; 2 Internal Medicine, University of Arkansas for Medical Sciences, Little Rock, USA; 3 Pulmonary and Critical Care, University of Arkansas for Medical Sciences, Little Rock, USA

**Keywords:** life threatening, medical emergency, electrocardiogram, pe, stemi

## Abstract

Pulmonary embolism is a common medical emergency and often life threatening but can be misdiagnosed frequently leading to fatal outcomes. Changes in electrocardiogram (ECG) are common in pulmonary embolism and rarely they can present with ST elevation. We here describe a 79-year-old woman who presented after a cardiac arrest and was found have ST-segment elevation on ECG with normal coronary angiogram while CT scan revealing pulmonary embolism.

## Introduction

Pulmonary embolism (PE) is a common medical emergency and often life threatening but can be misdiagnosed frequently leading to fatal outcomes, thus rapid diagnosis and treatment are essential in improving prognosis. Changes in electrocardiogram (ECG) are quite common in PE and can range from the most common and least specific; sinus tachycardia to the most specific and less frequent (S1Q3T3 pattern). We here describe a 79-year-old woman who presented after a cardiac arrest and was found have ST-segment elevation on ECG with normal coronary angiogram and massive PE.

## Case presentation

A 79-year-old woman was brought to the hospital after a cardiac arrest. Earlier that day she complained of feeling very hot and short of breath to the family but no chest pain. She has a history of hypertension, diabetes mellitus and umbilical hernia. She underwent surgery for incarcerated hernia about six weeks ago.

**Figure 1:**
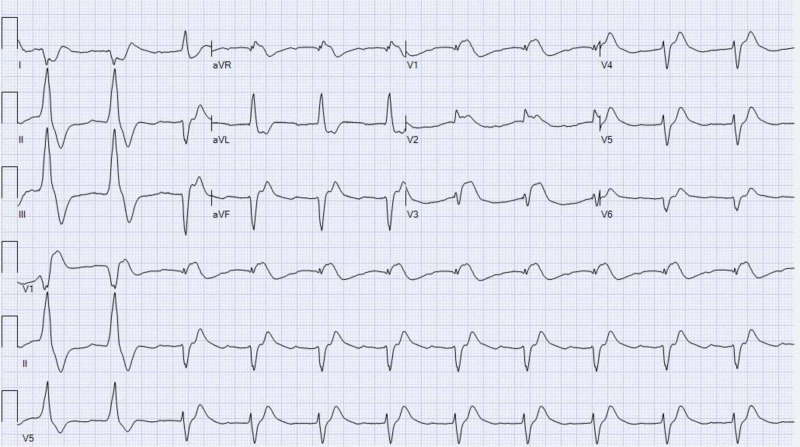
ECG showing normal sinus rhythm with ST-segment elevation in precordial leads V1-V3 with reciprocal ST-segment depression in lead I and aVL.

En route to the hospital, the patient had multiple episodes of cardiopulmonary arrest and return of spontaneous circulation (ROSC) was eventually achieved. Twelve lead ECGs on admission showed ST-segment elevation in leads V1-V3 (Figure 1), she underwent an emergent coronary angiogram that revealed normal coronary arteries.

**Figure 2:**
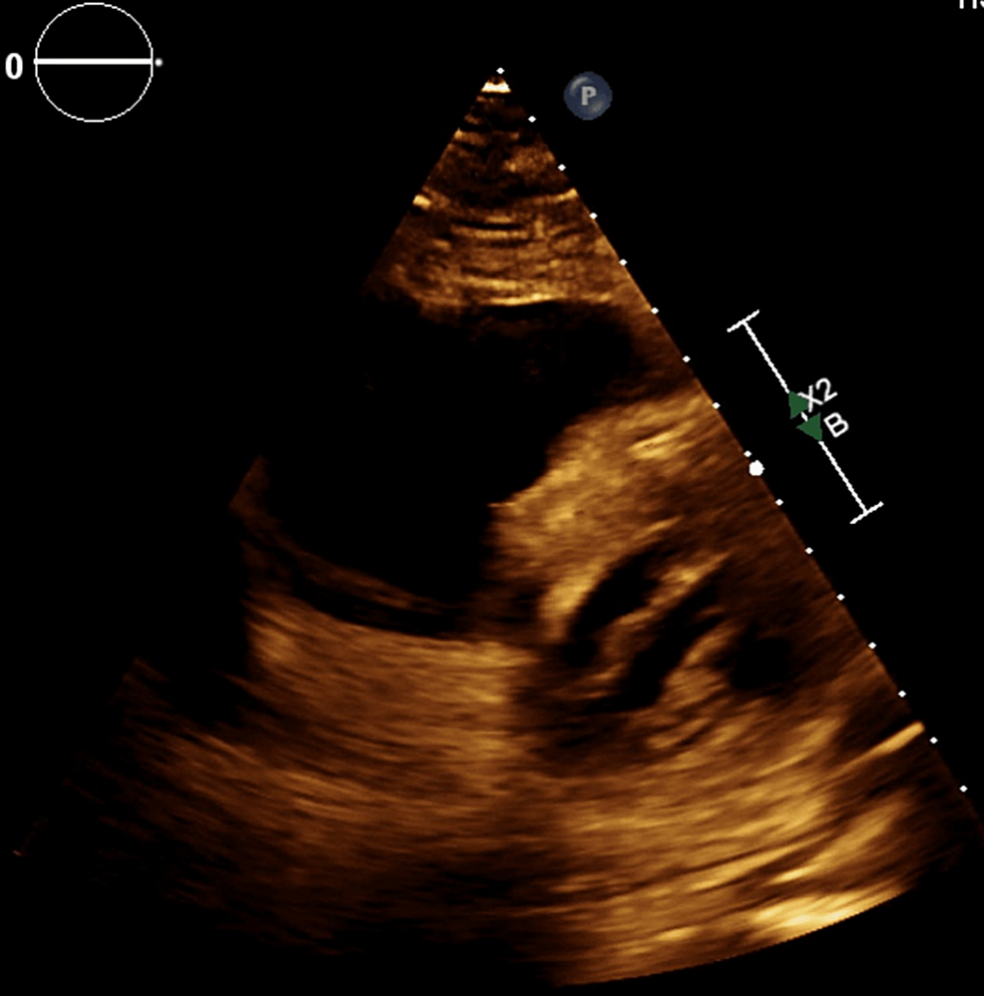
Echocardiogram showing dilated right ventricle.

The patient was intubated and placed on mechanical ventilation. She was started on norepinephrine, phenylephrine, and epinephrine for blood pressure support. The abdomen had a midline healing incision. Initial laboratory work was significant for elevated white blood cell count at 19.92 K/µL, glucose at 556 mg/dL, lactate at 16 mmol/L (normal range 0.5-2.2 mmol/L), BNP at 2,429 pg/mL (normal range <100 pg/mL), and elevated troponin at 2.98 ng/mL (normal range is less than 0.04 ng/mL). Computed tomography (CT) chest was significant for moderate to large burden of pulmonary emboli involving the right upper lobe, right lower lobe, left upper lobe, and left lower lobe segmental branches with the enlarged pulmonary trunk. CT head showed diffuse cerebral edema with cisternal effacement and herniation. Transthoracic echocardiogram showed an ejection fraction of 70% with flattened ventricular septum indicating right ventricle (RV) pressure overload. The RV was dilated with grossly reduced systolic function, the right atrium was mildly dilated and had a medium-sized papillary mobile mass possibly a thrombus, and the posterior leaflet of the mitral valve had an 11x14 mm echo dense mass that possible present old vegetation (Figure 2).

**Figure 3:**
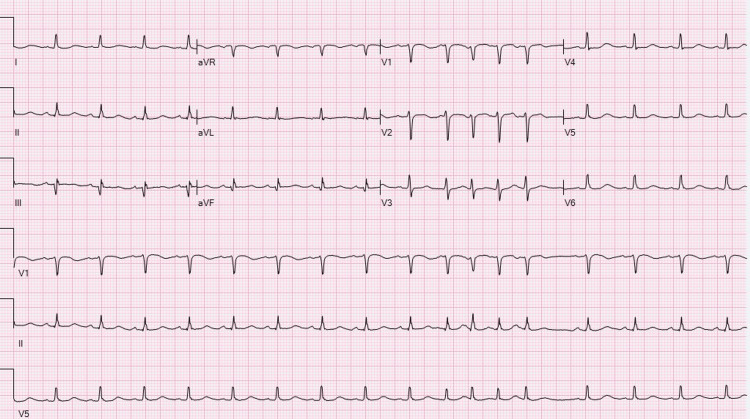
ECG showed resolution of the prior ST segment elevation. ECG, electrocardiogram.

As the patient had recent surgery and thrombolysis was contraindicated, the decision was made to start the patient on heparin infusion with a target PTT of 70-90. She was also started on hypothermia protocol and hypertonic saline was administrated for cerebral edema and impending herniation. Twelve lead ECGs after 48hours showed resolution of the ST segment elevation (Figure 3). During the hospital stay, the patient did not show any neurological improvement and she expired after a cardiopulmonary arrest.

## Discussion

PE is a common and possibly fatal diagnosis. Clinical presentation is widely variable and can range from asymptomatic to cardiopulmonary arrest [1]. Physicians should have a high index of suspicion. Rapid diagnosis and initiation of treatment are imperative as this disease is associated with increased hospital mortality that can reach up to 25% in patients presenting with shock and 65% for patients that require cardiopulmonary resuscitation [2].

Massive PE is defined as a PE that results in hypotension or shock; hypotension is defined as a drop in systolic pressure less than 90 or a drop of 40 or more from baseline for more than 15 minutes or hypotension requiring vasopressor support that is not explained by other causes [3].^ ^Diagnosis is typically achieved by a high index of suspicion based on the clinical presentation and a CT scan of the chest with contrast is confirmatory for PE.

Here, we presented a case of massive PE with an ECG showing ST elevations mimicking a myocardial infarction which was ruled out with normal coronaries by angiography. PE can present with a wide range of ECG changes with most of them being non-specific. The most common ECG finding is tachycardia and non-specific ST and T-wave changes [4].^ ^Arrhythmias including atrial fibrillation or atrial tachycardia and conduction delays including right bundle branch block commonly occurs [5].^ ^Despite the many proposed ECG findings with PE, ST segment elevation is a rare finding.

The ST-segment elevation is commonly associated with myocardial infarction; however, few other conditions can cause this finding. These conditions include but are not limited to Prinzmetals angina, ascending aortic dissection with extension to the right coronary artery, and Stage 1 of pericarditis which presents with diffuse ST-segment elevation. ST elevation can present chronically with early repolarization, left ventricular hypertrophy, Brugada syndrome, and left ventricular aneurysm.

ST-segment elevation in anteroseptal precordial leads is a rare but previously defined ECG finding [6].^ ^The mechanism for the ST elevations in the case of PE is not well known and many mechanisms have been proposed. One theory is that right ventricular strain caused by the pulmonary emboli leads to a mismatch between perfusion and oxygen supply and possibly leads to coronary vasospasms. Other proposes that Paradoxical embolization can occur especially with patent foramen ovale in the setting of PE leading to coronary occlusion [7]. catecholamine surge induced by severe hypoxemia leading to increase myocardial work is another possible mechanism [8].^ ^The echocardiogram in our case showed right ventricular overload and the coronary angiogram did not show any occlusion of the coronary arteries.

## Conclusions

In conclusion, ECG changes in PE are frequent but are highly variable. ST elevation in ECG points toward myocardial infarction most of the time. However, depending on the clinical scenario other etiologies such as pericarditis, takotsubo cardiomyopathy and in rare cases PE must be considered. Delay in diagnosis and treatment might lead to serious complications; thus, a high index of suspicion is needed. A bedside echocardiogram might provide early clues if right heart strain is evident as was seen in our case.
